# Biomarker profiling to determine clinical impact of microRNAs in cognitive disorders

**DOI:** 10.1038/s41598-024-58882-2

**Published:** 2024-04-09

**Authors:** Weijie Zhai, Meng Zhao, Chunxiao Wei, Guimei Zhang, Yiming Qi, Anguo Zhao, Li Sun

**Affiliations:** 1 Department of Neurology and Neuroscience Center, The First Hospital of Jilin University, Jilin University, Xinmin Street 1#, Changchun, 130021 China; 2 Department of Neurology, Cognitive Center, The First Hospital of Jilin University, Jilin University, Changchun, China; 3 Department of Urology, Dushu Lake Hospital Affiliated to Soochow University, Medical Center of Soochow University, Suzhou Dushu Lake Hospital, Suzhou, 215000 China

**Keywords:** Alzheimer’s disease, Bioinformatics, Cognitive impairment, Dementia, Post-stroke cognitive impairment, MicroRNA, Microbial communities, Immunology, Microbiology, Biomarkers, Neurology

## Abstract

Alzheimer’s disease (AD) and post-stroke cognitive impairment (PSCI) are the leading causes of progressive dementia related to neurodegenerative and cerebrovascular injuries in elderly populations. Despite decades of research, patients with these conditions still lack minimally invasive, low-cost, and effective diagnostic and treatment methods. MicroRNAs (miRNAs) play a vital role in AD and PSCI pathology. As they are easily obtained from patients, miRNAs are promising candidates for the diagnosis and treatment of these two disorders. In this study, we performed complete sequencing analysis of miRNAs from 24 participants, split evenly into the PSCI, post-stroke non-cognitive impairment (PSNCI), AD, and normal control (NC) groups. To screen for differentially expressed miRNAs (DE-miRNAs) in patients, we predicted their target genes using bioinformatics analysis. Our analyses identified miRNAs that can distinguish between the investigated disorders; several of them were novel and never previously reported. Their target genes play key roles in multiple signaling pathways that have potential to be modified as a clinical treatment. In conclusion, our study demonstrates the potential of miRNAs and their key target genes in disease management. Further in-depth investigations with larger sample sizes will contribute to the development of precise treatments for AD and PSCI.

## Introduction

Dementia encompasses a range of syndromes characterized by a decline in cognitive and executive abilities, significantly affecting daily life^1^. Globally, over 50 million people suffer from dementia, and the number of patients is projected to rise to 152 million by 2050^2^. In younger individuals, they are often associated with severe brain trauma and infections. Among the elderly, common causes include Alzheimer's disease (AD), post-stroke cognitive impairment (PSCI), vascular dementia (VaD), and dementia with Lewy bodies (DLB). Dementia prevalence has seen an increase in the older population (≥ 60 years old), with the age-standardized rate ranging between 5 and 7%. Notably, this rate approximately doubles every 5 years^3^. Unfortunately, the diagnosis and treatment of both AD and PSCI present major challenges. Genetic research is crucial for understanding AD/PSCI etiology and paving the way for novel therapeutic options.

Approximately 60–80% of projected dementia cases are attributed to AD^2^. Because pathological changes in AD occur decades before the symptoms are detectable, early diagnosis is key to effective treatment. The primary characteristic of AD is impaired clearance of β-amyloid (Aβ), and the formation and accumulation of hyperphosphorylated tau protein tangles. These pathological features arise from abnormal processing of amyloid precursor protein by β-/γ-secretases, leading to the production and aggregation of Aβ40 and Aβ42 monomers^4,5^. Hyperphosphorylated tau proteins contribute to synaptic disruption, neuronal dysfunction, and development of neurofibrillary tangles^6^. In addition, cerebrovascular lesions, oxidative stress (OS), neuroinflammation, and abnormal glucose metabolism all combine to play complex roles in AD initiation and progression^7^. Currently, AD diagnosis relies on neuropsychological and neuroimaging assessments, biomarker-based lumbar puncture, and positron emission tomography (PET). These methods are both costly and invasive, limiting their clinical implementation. Furthermore, available treatments currently can only address symptoms, while they can improve quality of life temporarily, therapies that halt or reverse disease progression have not been developed, despite over 2000 clinical trials testing various targets and treatment strategies^8^.

Research on non-coding RNAs (ncRNAs) have advanced rapidly in recent years, especially with the development of high-throughput sequencing, whole-gene sequencing, and bioinformatics. Of particular interest are microRNAs (miRNAs), 18–21 nucleotides that regulate the expression of downstream genes through cleaving or post-transcriptionally inhibiting target mRNAs. Through this process, miRNAs modulate numerous intracellular and extracellular signaling pathways in the neocortex, hippocampus, and limbic systems. Specifically, miRNAs are involved in regulating tight connections in the blood–brain barrier (BBB), affecting neuronal growth, proliferation, and differentiation. They also participated in the regulation of synaptic homeostasis and plasticity^9–11^. As the CNS ages, miRNA abundance alters in accordance with neural development and differentiation^12–14^. In-depth analysis of miRNAs is hoped to uncover novel pathogenic pathways or pathogenic factors in known pathways that can benefit AD therapies.

Compared with PET or protein analysis of cerebrospinal fluid, the major advantage of miRNAs is their lack of invasiveness while providing high volumes of data. A recent systematic review cross-validated 250 miRNAs associated with AD, highlighting 10 miRNAs (miR-107, miR-26b, miR-30e, miR-34a, miR-485, miR-200c, miR-210, miR-146a, miR-34c, and miR-125b) in the peripheral blood of patients^15^. These were hypothesized to be deregulated 20 years before clinical symptom onset^15^ and are associated with major biological processes, including immune system function, cell cycle regulation, gene expression, neuron growth factor signaling, Wnt signaling, cellular senescence, and Rho GTPase activity. Of these, the involvement of miRNAs in autophagy^16^ and neuronal mitochondria are particularly relevant to AD etiology^17^. Notably, miR-455-3p upregulation enhances synaptic activity, suggesting a potential neuroprotective role^18^. In contrast, miR-34a dysregulation causes dysfunction in resting presynaptic and postsynaptic activity^19^. Other specific links between miRNAs and AD include miR-29c-3p, miR-193b, miR-132, miR-103, and miR-181c regulating Aβ activity; miR-181c, miR-31, miR-93^20^, and miR-146^21^ modulating microglial inflammatory response in the CNS; as well as miR-125b^22^, miR-26b, miR-483-5p, and miR-502-3p influencing tau protein activity and NFT formation^23^. Additionally, miR-532-5p targets EPHA4 to ameliorate BBB damage^24^, while miR-126 targets EFHD2 to promote the formation and modulation of contextual fear memory in patients with AD^25^. The available data all point to the importance of miRNAs in influencing AD onset and symptoms.

To be classified as PSCI, patient performance on a multidomain neuropsychological assessment or the Montreal Cognitive Assessment must be lower than the fifth percentile of local normative data in at least one cognitive domain^26^. The condition is a common consequence of ischemic stroke that causes long-term disability and lowers quality of life^27,28^. Among dementias, PSCI has the second highest incidence after AD^29^. Pooled data analyses indicated a prevalence of 38%^30^ PSCI and 18.4%^31^ post-stroke dementia in the first year after stroke^32^. Although PSCI is largely attributable to vascular factors, neurodegenerative changes are also common in elderly patients and can interact with PSCI^33^. A pivotal prerequisite for PSCI is cerebral small vessel disease (CSVD), characterized by arteriolar sclerosis, lacunar infarction, cortical and subcortical microinfarction, as well as pathological changes to white matter (including myelin loss and axonal abnormalities)^34^. Post-stroke inflammation may also play a role in amyloid deposition during PSCI development^35^.

Diagnosis of PSCI currently relies on clinical symptoms, neuropsychology, and neuroimaging. Deep learning (DL)-based cognitive evaluation using FDG-PET is an important technique for assessing cognitive dysfunction^36^. Infarct location and stroke characteristics can predict PSCI independently of demographic and vascular risk factors, particularly the presence of multiple acute infarcts, total infarct volume, and left cerebral hemispheric location^27,37^. In terms of PSCI biomarkers, numerous potential targets have been proposed, including indicators of genetic polymorphisms, biomarkers in the cerebrospinal fluid and plasma, inflammatory mediators, and peripheral miRNAs^38^. However, thus far, none can discriminate vulnerable patients robustly.

Research using TaqMan Low-Density Arrays and single TaqMan assays have demonstrated that miR-10b, miR29a-3p, and miR-130b-3p are downregulated in the plasma of patients with PSCI and AD, especially miR-130b-3p in AD^39^. Experiments rats showed that intravenous (iv) injections of miR-20a-3p improved acute stroke outcomes in male and female rats, while also ameliorating stroke-induced cognitive decline in female rats^40^. Additionally, intracerebroventricular (ICV) injection of miR-210-5p agonist aggravated synaptic loss and cognitive impairment in VD rats, specifically through regulating synaptosomal-associated protein of 25 KDa^41^. Serum miRNA profiling of patients and healthy controls identified seven miRNAs (miR-1228-5p, miR-1268a, miR-1268b, miR-4433b-3p, miR-6090, miR-6752-5p, and miR-6803-5p) that could predict the risk of cerebrovascular disorders before stroke onset^42^. Furthermore, acupuncture appeared to attenuate inflammation-related PSCI through inhibiting the miR-93-mediated TLR4/MyD88/NF-κB signaling pathway^20^. Similarly, the TNFα–miR-501-3p–ZO-1 axis modulates BBB to affect working memory deficits and white matter lesions^43^. Overall, therapies based on miRNAs may provide novel insights into inhibiting PSCI progression.

Although numerous preclinical studies involving miRNA therapeutics have been conducted, only a few have moved into clinical development. One of the biggest challenges in developing these treatments is the identification of suitable miRNAs and their targets for each disease type^44–46^. In this study, we aimed to provide more candidates through high-throughput sequencing, screening, and functional analysis of differentially expressed miRNAs (DE-miRNAs). Our findings should expand current understanding of miRNAs related to cognitive decline and provide options for further research on miRNA-based treatment.

## Results

### Characterization of participants

Medical histories did not differ across the AD, PSCI, post-stroke non-cognitive impairment (PSNCI), and normal control (NC) groups. Sex ratios, exhibited between-group variation (*P* < 0.05), but pair-to-group comparisons after Bonferroni correction showed no significant differences between pairs (Table S1). Upon assessment, we identified between-group differences in the MMSE, MoCA, and various cognitive domains (*P* < 0.05) (Table 1).

**Table 1:** Neuropsychological assessment in each group: Alzheimer’s disease (AD), post-stroke cognitive impairment (PSCI), post-stroke non-cognitive impairment (PSNCI), and normal control (NC).

	AD	PSCI	PSNCI	NC	*P*
MMSE (score)	16.5 ± 7.1*	23.5 ± 2.9*^^^	28.3 ± 1.5	28.7 ± 1.5	0.003
Orientation to time (score)	2.0 (1.0–3.5)*	4.0 (2.5–4.3)^^^	5.0 (4.0–5.0)	5.0 (4.0–5.0)	0.010
Orientation to place (score)	3.5 (2.8–4.3)*	5.0 (3.8–5.0)	5.0 (5.0–5.0)	5.0 (5.0–5.0)	0.005
Memory (score)	2.5 (1.78–3.0)	3.0 (2.0–3.0)	3.0 (3.0–3.0)	3.0 (3.0–3.0)	0.084
Attention and calculation (score)	1.0 (0.8–3.50*	3.5 (2.5–5.0)	5.0 (4.8–5.0)	4.5 (3.5–5.0)	0.026
Recall (score)	0.0 (0.0–1.3)*^†^	2.0 (1.0–2.3)*	2.0 (2.0–3.0)	3.0 (2.8–3.0)	0.002
Language (score)	5.5 (2.8–7.0)	6.0 (5.0–6.3)*	7.0 (6.0–7.0)	7.0 (7.0–7.0)	0.022
Write (score)	1.0 (0.8–1.0)	1.0 (1.0–1.0)	1.0 (0.8–1.0)	1.0 (1.0–1.0)	0.554
Structural (score)	0.0 (0.0–0.3)	1.0 (0.0–1.0)	1.0 (0.8–1.0)	1.0 (0.8–1.0)	0.062
MoCA (score)	11.8 ± 5.4*	16.5 ± 4.4*^^^	25.0 ± 1.7	25.7 ± 2.7	< 0.001
Visuospatial and executing (score)	1.0 (0.8–2.3)*	2.0 (0.8–3.3)*	3.0 (3.0–5.0)	5.0 (3.8–5.0)	0.003
Naming (score)	3.0 (1.0–3.0)	3.0 (2.0–3.0)	3.0 (3.0–3.0)	3.0 (2.0–3.0)	0.449
Attention (score)	1.5 (1.0–2.0)	1.0 (1.0–2.0)	2.0 (2.0–2.0)	2.0 (2.0–2.0)	0.020
Read on (score)	0.0 (0.0–1.0)	1.0 (0.0–1.0)	1.0 (1.0–1.0)	1.0 (1.0–1.0)	0.025
Calculation (score)	1.0 (0.8–2.3)	2.5 (1.0–3.0)	3.0 (3.0–3.0)	2.5 (1.8–3.0)	0.031
Verbal fluency (score)	0.5 (0.0–1.0)*	2.0 (0.8–2.3)*^	3.0 (3.0–3.0)	3.0 (2.8–3.0)	< 0.001
Abstraction (score)	0.5 (0.0–1.25)	1.0 (0.0–1.25)	2.0 (1.75–2.0)	1.0 (0.75–2.0)	0.053
Delayed recall (score)	0.0 (0.0–0.0)*	0.0 (0.0–1.25)*	1.5 (0.75–2.25)	3.5 (2.5–4.0)	0.001
Direction (score)	3.0 (1.0–3.8)*	5.0 (3.5–5.3)*^	6.0 (5.8–6.0)	6.0 (5.8–6.0)	0.006
ADL (score)	30.5 (23.5–33.0)*	22.0 (20.0–24.0)	20.0 (20.0–21.8)	20.0 (20.0–20.0)	0.002
CDR (score)	1.5 (1.0–3.0)*	1.0 (1.0–1.3)*^	0.0 (0.0–0.1)	0.0 (0.0–0.1)	< 0.001
HAMD (score)	2.5 (1.0–5.8)	3.0 (2.3–4.8)	3.0 (0.0–6.5)	3.5 (0.0–5.5)	0.995
HAMA (score)	3.5 (2.8–6.0)	0.5 (0.0–4.8)	2.0 (1.0–2.5)	2.0 (0.0–10.3)	0.263

†*P* < 0.05: compared with PSCI group; ^*P* < 0.05: compared with the PSNCI group; *P <0.05: compared with NC group.

### Bioinformatics analysis of AD and control (NC)

**Figure 1:**
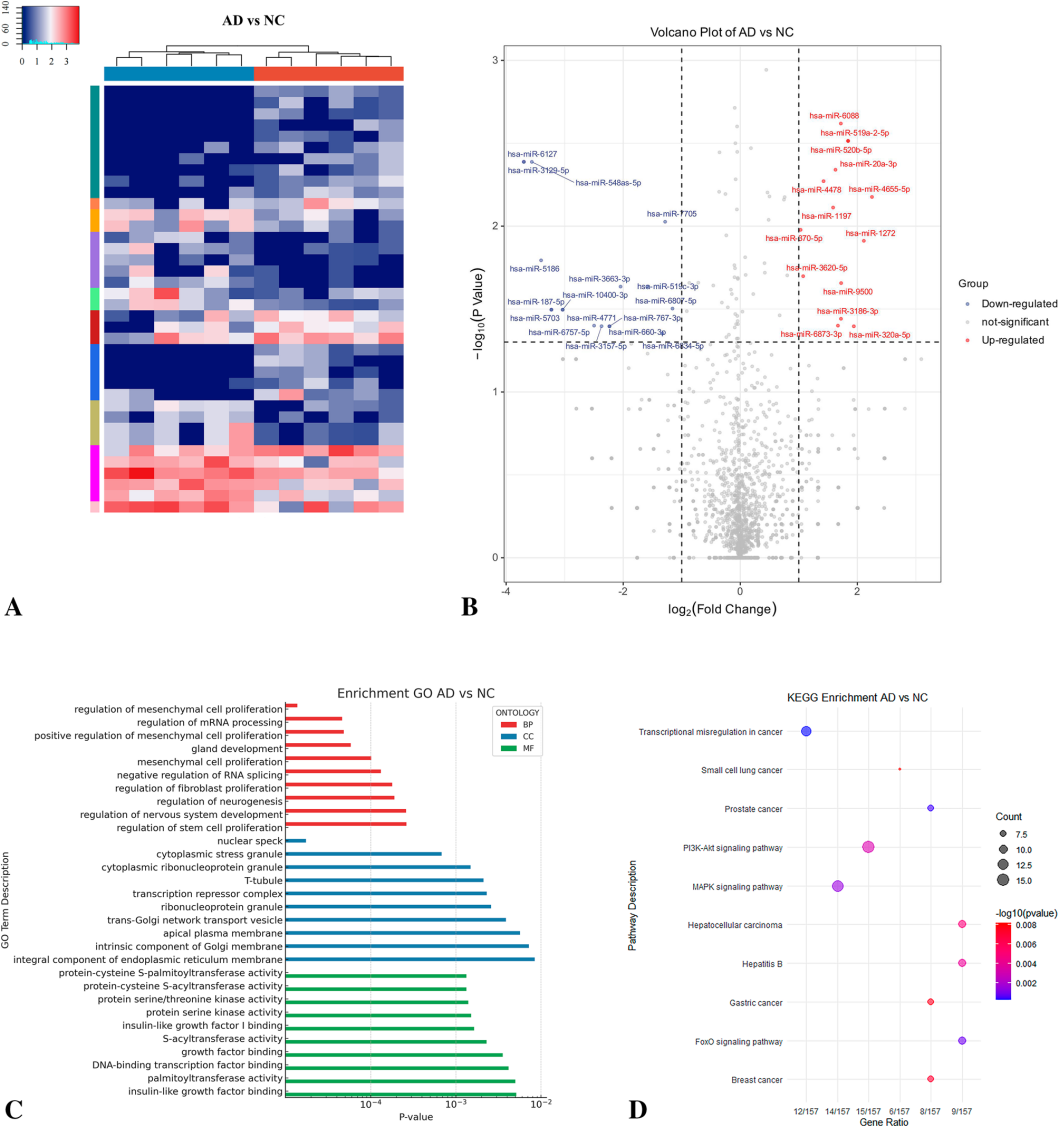
Bioinformatics analysis comparing Alzheimer’s disease (AD) and normal controls (NC). (**A**) Sequencing cluster map between the AD and NC groups. Different colors indicate relative expression levels (log2-transformed). Blue, below-mean expression; red, above-mean expression. The colored bar at the top of the panel represents participating groups (blue represents AD and red represents NC). The colored bar on the right side of the panel indicates divisions based on K-means. (**B**) Volcano plot of DE-miRNAs between AD and NC groups. On the x-axis, the dotted line indicates DE-miRNAs that satisfied the |log2FC|≥ 1 cut-off. On the y-axis, the dotted line indicates DE-miRNAs that satisfied *P* < 0.05. Red, highly expressed; purple, lowly expressed. (**C**) Gene Ontology (GO) enrichment results between AD and NC. *P* values are on the X-axis (significance of associations between gene sets and GO terms). The Y-axis displays GO terms. (**D**) The KEGG^47–49^ pathway analysis between AD and NC. Gene ratio is proportion of genes in a pathway to total genes analyzed (x-axis). Signaling pathways are on the y-axis. Bubble size indicates the number of genes enriched in a given pathway. Bubble color reflects significance level (bluer is smaller). *P*-values are log-transformed.

We identified 2263 common miRNAs between the AD and NC groups (Fig. 1A; Table S2). With thresholds of *P* < 0.05 and |log2FC|≥ 1, we found 14 upregulated and 17 dysregulated DE-miRNAs (Fig. 1B). Of these 31 DE-miRNAs, relevant data for hsa-miR-320a-5p, hsa-miR-519a-2-5p, hsa-miR-520b-5p, and hsa-miR-10400-3p were not retrieved from the TargetScan Human database. The final analysis included 27 DE-miRNAs that targeted 384 genes.

After gene set enrichment analyses (GSEA), we restricted our display to the top 10 Gene Ontology (GO) terms per category and the principal 10 Kyoto Encyclopedia of Genes and Genomes (KEGG) pathways (Fig. 1C,D; Table S3 and S4). Target genes were significantly enriched in the biological processes of mesenchymal and fibrocyte proliferation, neurogenesis, mRNA processing, stem cell proliferation, and nervous system development. They were located in multiple organelles, including cytoplasmic stress particles, transcription inhibitor complexes, nuclide spots, nuclear specks, integral components of the endoplasmic reticulum membrane, and apical plasma membrane. In terms of molecular function, target genes were involved in protein cysteine S-acyltransferase activity, protein serine muscle enzyme activity regulation, insulin-like growth factor 1, and growth factor-binding hormone. The results of KEGG analysis suggested that target genes mainly participated in the phosphatidylinositol 3-kinase (PI3K)-Akt, MAPK, and FoxO signaling pathways, as well as in small cell lung cancer and prostate cancer (Fig. 1D).

**Figure 2:**
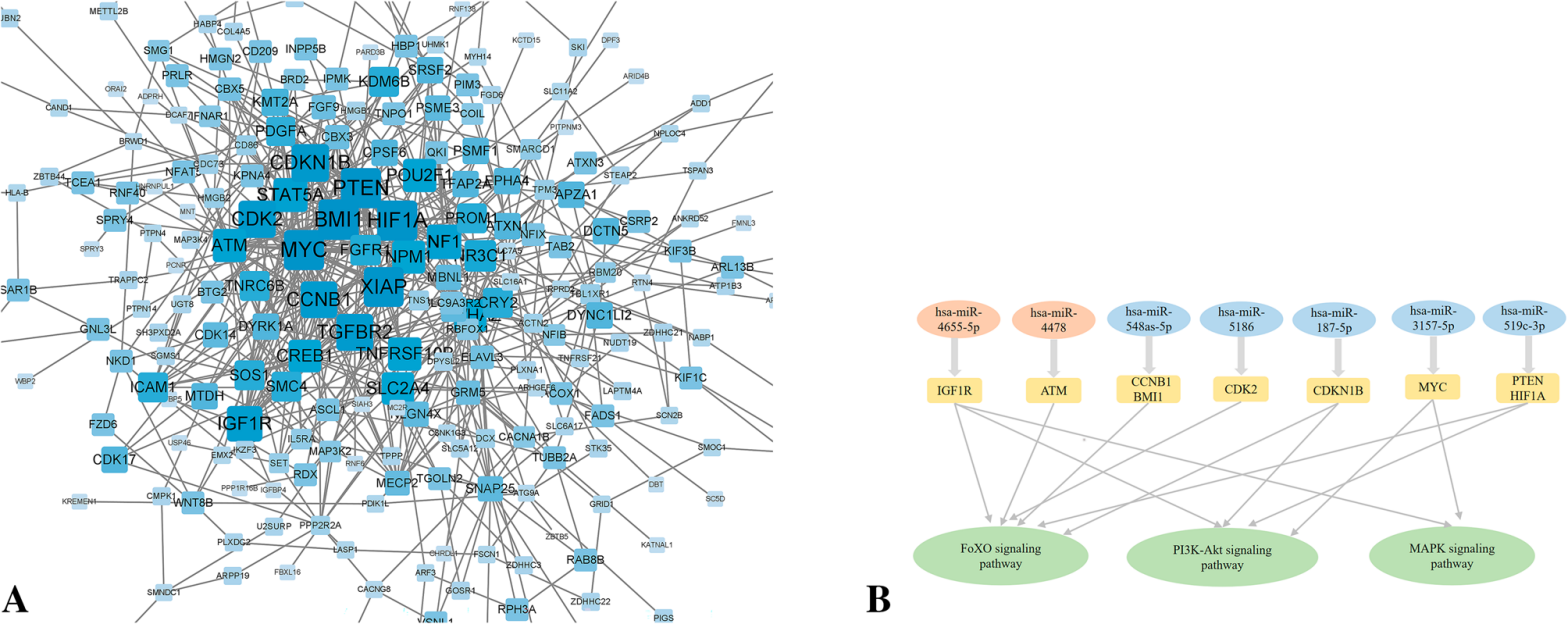
Target genes of DE-miRNAs between AD and NC. (**A**) STRING visualization of the protein–protein interaction (PPI) network. The darker the color, the larger the font, and the larger the background area, the greater the weight of this gene in the PPI network and its correlation with other genes. (**B**) DE-miRNAs plus their key target genes.

We then constructed a protein–protein interaction (PPI) network of DE-miRNA target genes, comprising 364 gene/protein nodes and 655 non-redundant interacting edges (Fig. 2A). Analysis in MOCDE and CytoHubba yielded nine key genes: *ATM*, *MYC*, *PTEN*, *CDKN1B*, *CCNB1*, *HIF1A*, *CDK2*, *BMI1*, and *IGF1R*. Analysis of interactions between DE-miRNAs and key genes (Fig. 2B) revealed that a single DE-miRNA has multiple targets, while one gene can affect several downstream signaling pathways.

### Bioinformatics analysis of AD and PSCI

**Figure 3:**
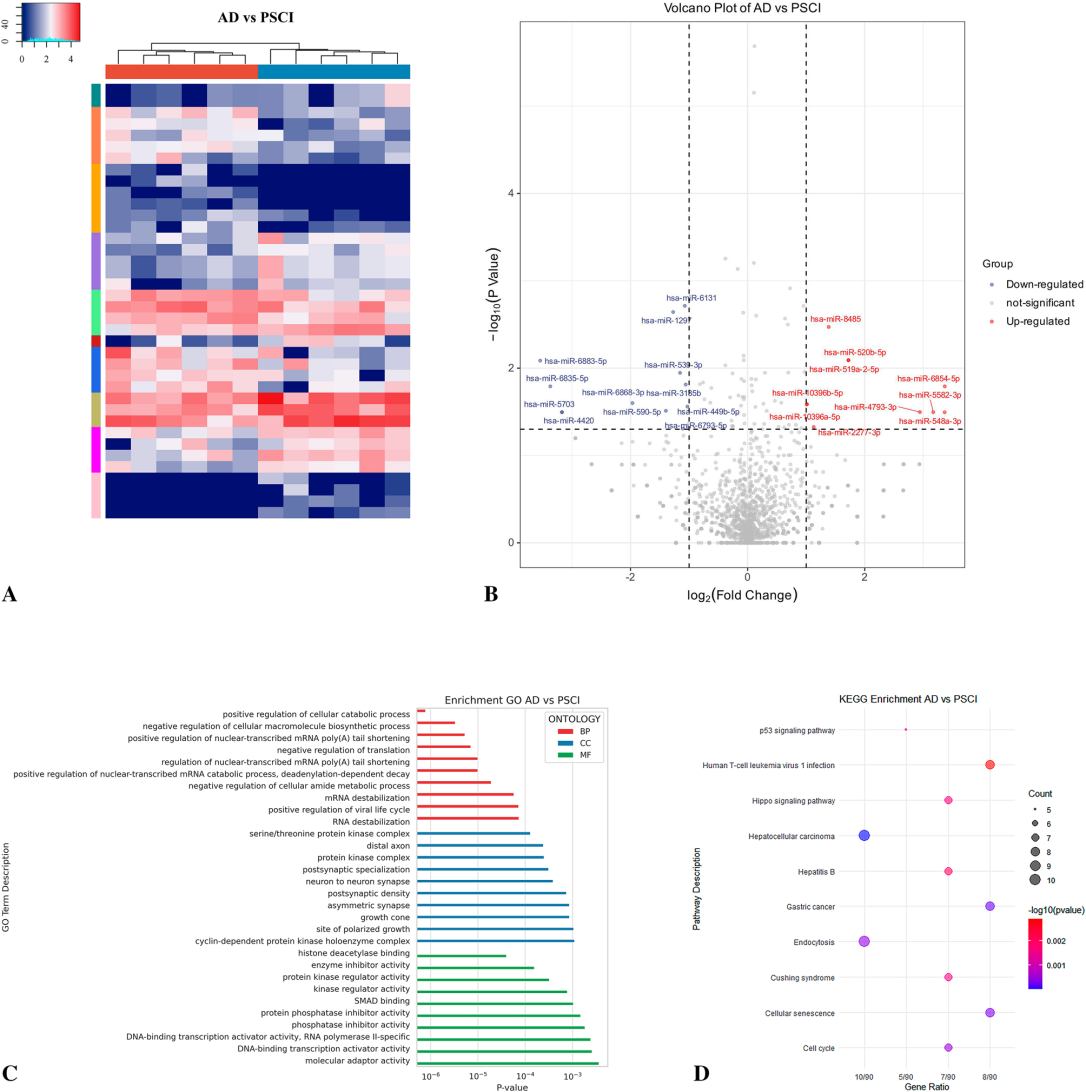
Bioinformatics analysis comparing Alzheimer’s disease (AD) and post-stroke cognitive impairment (PSCI). (**A**) Sequencing cluster map between the AD and PSCI groups. Different colors indicate relative expression levels (log2-transformed). Blue, below-mean expression; red, above-mean expression. The colored bar at the top of the panel represents participating groups (blue represents AD and red represents PSCI). The colored bar on the right side of the panel indicates divisions based on K-means. (**B**) Volcano plot of DE-miRNAs between AD and PSCI groups. On the x-axis, the dotted line indicates DE-miRNAs that satisfied the |log2FC|≥ 1 cut-off. On the y-axis, the dotted line indicates DE-miRNAs that satisfied *P* < 0.05. Red, highly expressed; purple, lowly expressed. (**C**) Gene Ontology (GO) enrichment results between AD and PSCI. *P* values are on the X-axis (significance of associations between gene sets and GO terms). The Y-axis displays GO terms. (**D**) The KEGG^47–49^ pathway analysis between AD and PSCI. Gene ratio is proportion of genes in a pathway to total genes analyzed (x-axis). Signaling pathways are on the y-axis. Bubble size indicates the number of genes enriched in a given pathway. Bubble color reflects significance level (bluer is smaller). *P*-values are log-transformed.

**Figure 4:**
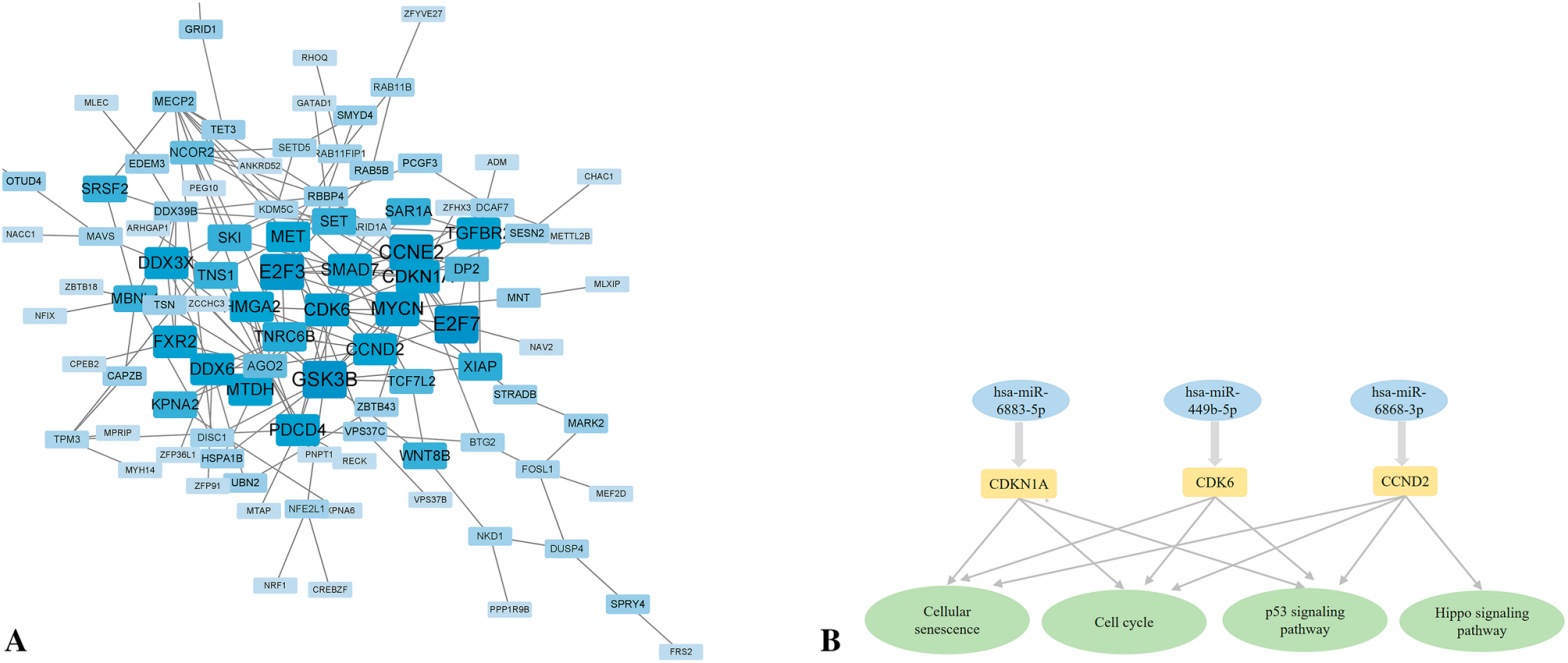
Target genes of DE-miRNAs between AD and PSCI. (**A**) STRING visualization of the protein–protein interaction (PPI) network. The darker the color, the larger the font, and the larger the background area, the greater the weight of this gene in the PPI network and its correlation with other genes. (**B**) DE-miRNAs plus their key target genes.

We identified 2242 miRNAs that were common across AD and PSCI groups (Table S2; Fig. 3A). Of these, 22 were DE-miRNAs (10 upregulated and 12 dysregulated) (Fig. 3B). Using biogenic analysis, we then conducted GSEA on DE-miRNA target genes (Table S5 and S6); the top 10 GO terms per category and top 10 KEGG pathways are shown (Fig. 3C,D). Target genes participate in the positive regulation of cellular catabolic processes, nuclear-transcribed mRNA poly(A) tail shortening and viral life cycle, negative regulation of cellular macromolecule biosynthetic processes, and cellular amide metabolic processes. Additionally, they are involved in the cellular components of growth cones, asymmetric synapses, neuron-to-neuron synapses, and postsynaptic specialization. Finally, the target genes play a role in regulating enzyme inhibitor activity, protein kinase activity, kinase activity, protein phosphatase inhibitor activity, DNA-binding transcription activator activity, and molecular adaptor activity. The results of KEGG analysis indicated that these genes are mainly involved in cell catabolism, senescence, and endocytosis, as well as the Hippo and p53 signaling pathways. Key target genes of these DE-miRNAs are CDKN1A, CDK6, and CCND2 (Fig. 4A,B).

### Bioinformatics analysis of PSCI and PSNCI

**Figure 5:**
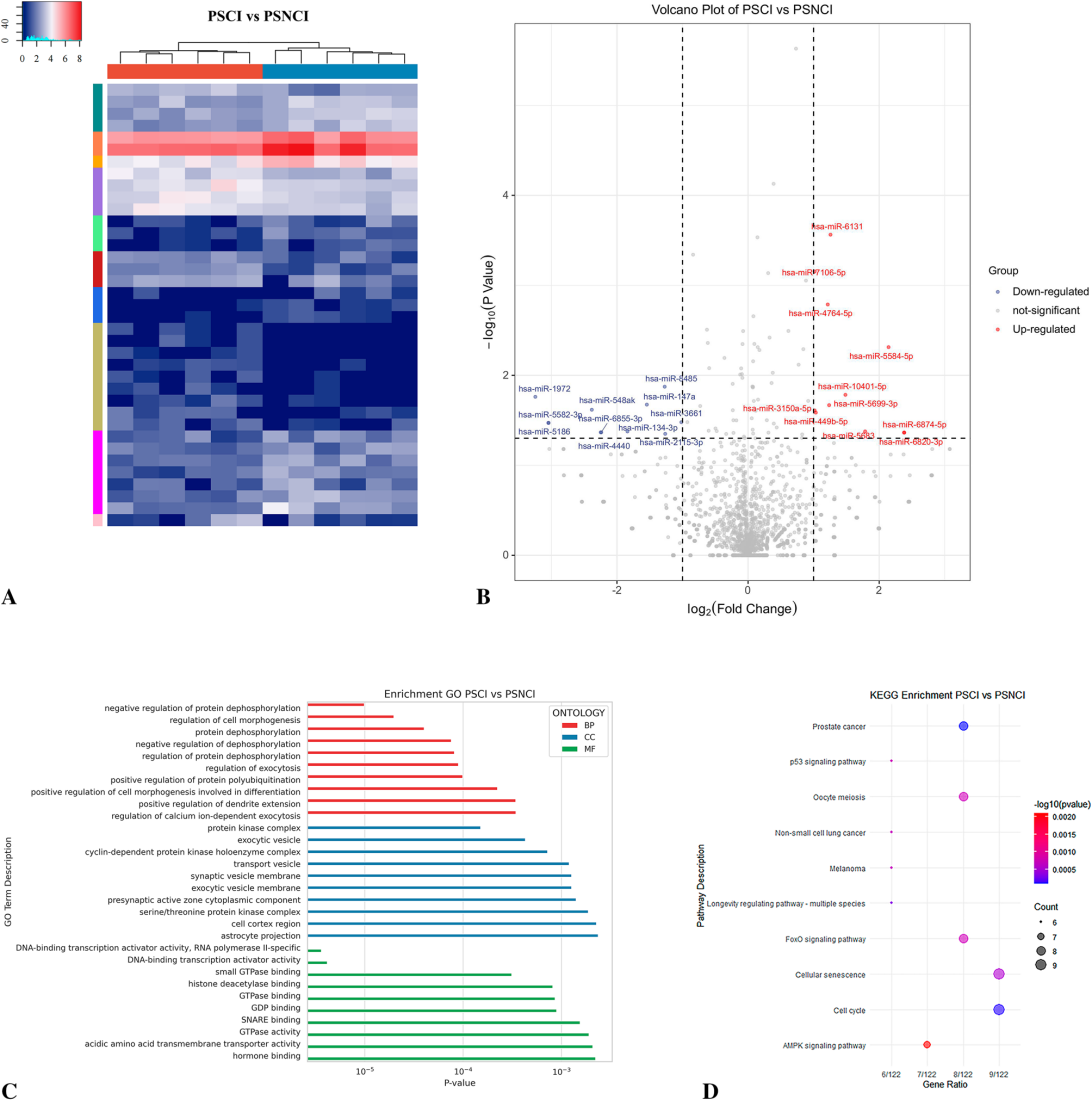
Bioinformatics analysis comparing post-stroke cognitive impairment (PSCI) and normal post-stroke non-cognitive impairment (PSNCI). (**A**) Sequencing cluster map between the PSCI and PSNCI groups. Different colors indicate relative expression levels (log2-transformed). Blue, below-mean expression; red, above-mean expression. The colored bar at the top of the panel represents participating groups (blue represents PSCI and red represents PSNCI). The colored bar on the right side of the panel indicates divisions based on K-means. (**B**) Volcano plot of DE-miRNAs between PSCI and PSNCI groups. On the x-axis, the dotted line indicates DE-miRNAs that satisfied the |log2FC|≥ 1 cut-off. On the y-axis, the dotted line indicates DE-miRNAs that satisfied *P* < 0.05. Red, highly expressed; purple, lowly expressed. (**C**) Gene Ontology (GO) enrichment results between PSCI and PSNCI. *P* values are on the X-axis (significance of associations between gene sets and GO terms). The Y-axis displays GO terms. (**D**) The KEGG^47–49^ pathway analysis between PSCI and PSNCI. Gene ratio is proportion of genes in a pathway to total genes analyzed (x-axis). Signaling pathways are on the y-axis. Bubble size indicates the number of genes enriched in a given pathway. Bubble color reflects significance level (bluer is smaller). *P*-values are log-transformed.

**Figure 6:**
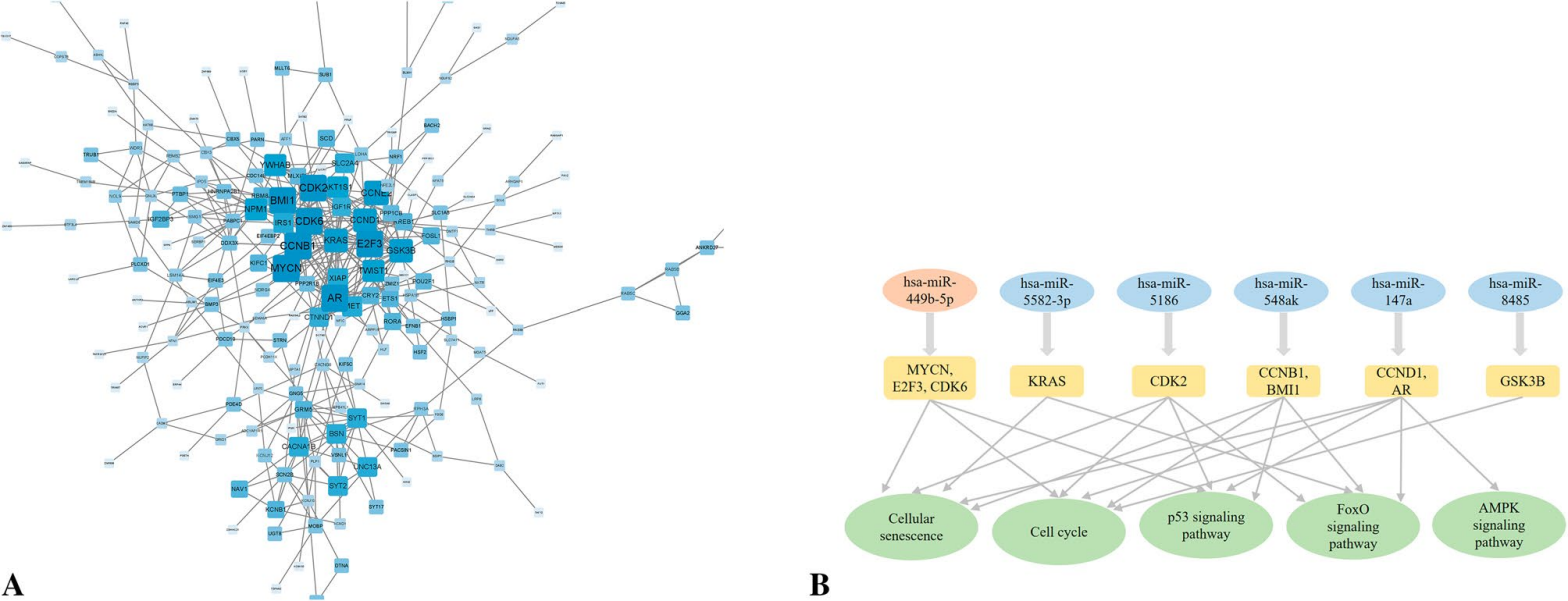
Target genes of DE-miRNAs between PSCI and PSNCI. (**A**) STRING visualization of the protein–protein interaction (PPI) network. The darker the color, the larger the font, and the larger the background area, the greater the weight of this gene in the PPI network and its correlation with other genes. (**B**) DE-miRNAs plus their key target genes.

We identified 2238 common miRNAs across the PCI and PSNCI groups (Table S2; Fig. 5A). Of these, 22 DE-miRNAs were found (11 upregulated and 11 dysregulated) (Fig. 5B), but data for hsa-miR-10401-5p was absent from the TargetScan Human database, resulting in 21 DE-miRNAs used for target gene prediction (Table S7 and S8). Only the top 10 GO terms and the top 10 KEGG pathways are displayed (Fig. 5C,D). The results of GO analysis showed that target genes were enriched in protein dephosphorylation-related biological progression. They are localized to the synaptic vesicle membrane, presynaptic active zone cytoplasmic component, and cell cortex region. Additionally, they participate in metabolic progresses related to small GTPase binding, GDP binding, GTPase activity, acidic amino acid transmembrane transporter activity, and hormone binding. Next, the results of KEGG analysis showed that target genes are involved in FoxO, AMPK, and P53 signaling pathways. Construction of a PPI network then identified *BMI1*, *CDK2*, *CCNB1*, *KRAS*, *CCND1*, *CDK6*, *MYCN*, *AR*, *E2F3*, and *GSK3B* as most important genes interacting with DE-miRNAs (Fig. 6A,B).

### Comparison of DE-miRNAs and target genes across three groups

**Figure 7:**
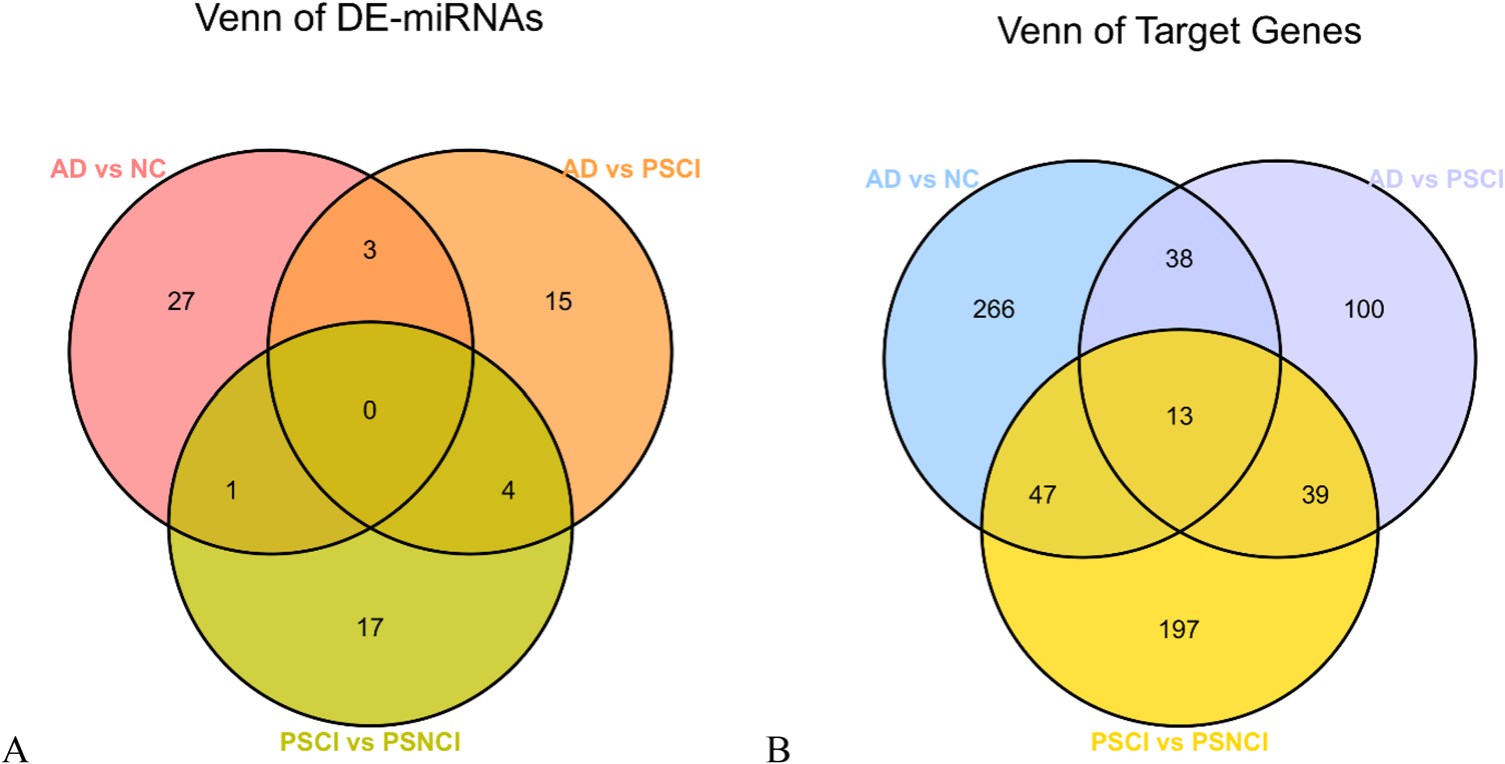
Venn diagrams representing DE-miRNAs and target genes common across all three groups with cognitive impairments. (**A**) DE-miRNAs between patients with different cognitive impairments (AD, PSCI, PSNCI) and healthy controls (NC). Red circle, AD and NC; orange circle, AD and PSCI; green circle, PSCI and PSNCI. (**B**) Differentially expressed target genes between patients with different cognitive impairments and healthy controls. Blue circle, AD and NC; purple circle, AD and PSCI; yellow circle, PSCI and PSNCI.

Three DE-miRNAs (hsa-miR-519a-2-5p, hsa-miR-520b-5p, hsa-miR-5703) were differentially expressed in both AD vs. NC and AD vs. PSCI. Four DE-miRNAs (hsa-miR-5582-3p, hsa-miR-8485, hsa-miR-6131, hsa-miR-449b-5p) were differentially expressed in AD vs. PSCI and PSCI vs. PSNCI. Only one DE-miRNA (hsa-miR-5186) was differentially expressed in AD vs. NC and PSCI vs. PSNCI (Fig. 7A; Table S9). Notably, no DE-miRNAs were common across all three groups, but target gene expression exhibited between-group variation (Fig. 7B; Table S10). Our analysis identified 38 differentially expressed target genes for AD vs. NC and AD vs. PSCI, 39 for AD vs. PSCI and PSCI vs. PSNCI, and 47 for AD vs. NC and PSCI vs. PSNCI. Thirteen target genes were differentially expressed across all three comparative pairs.

## Discussion

By collecting data from patients with different dementia subtypes and comparing plasma miRNA expression, our research successfully identified novel miRNAs related to AD and PSCI. Moreover, we linked those miRNAs to their target genes, providing considerable clarity on the molecular mechanisms underlying AD and PSCI.

With the development of genome-wide association studies (GWAS) and single-cell sequencing technologies, miRNAs are increasingly relevant as biomarkers for non-invasive diagnosis and treatment. Different miRNA combinations have been investigated for their suitability in diagnosing cognitive disorders, including AD and PSCI^50^. Similarly, our target gene prediction and GSEA identified 14 DE-miRNAs. Of these, hsa-miR-548as-5p, hsa-miR-5186, and hsa-miR-449b-5p were differentially expressed across all three patient groups. Functional analysis with KEGG demonstrated that DE-miRNAs participate in AD and PSCI progression through their target genes in the cell cycle, cell senescence processes, as well as FoxO, PI3K, Hippo, and p53 signaling pathways. We also identified several novel DE-miRNAs, including hsa-miR-548as-5p, hsa-miR-519c-3p, hsa-miR-6883-5p, hsa-miR-5186, hsa-miR-548ak, and hsa-miR-147a. The remaining DE-miRNAs have been implicated in infectious diseases^51^, endocrine diseases^52^ and neoplastic diseases^53–56^, but not in CNS diseases or any cognitive disorders. Notably, changes in cellular developmental pathways do not yield the same outcome in every disease; in cancers, they cause rapid cell growth and proliferation, whereas in CNS disorders, they accelerate neuron aging and death. Understanding the specific characteristics of neoplastic diseases will be useful for producing novel interventions and targets in AD and PSCI^57^.

We compared DE-miRNAs and target genes across three pairs of groups (AD vs. NC, AD vs. PSCI, and PSCI vs. PSNCI) to better understand which miRNA expression patterns are common or unique across disease states. None of the identified DE-miRNAs were present in all three groups. However, has-miR-5582-3, hsa-miR-8485, hsa-miR-6131, hsa-miR-449b-5p, and hsa-miR-5186 were important for both AD and PSCI development, while hsa-miR-519a-2-5p, hsa-miR-520b-5p, and hsa-miR-5703 were unique to AD, allowing for differential diagnosis from NC and PSCI. Furthermore, we identified 13 target genes (*STK35*, *GRID1*, *ZNF268*, *PARP11*, *RAB5B*, *PDE7A*, *RNF40*, *KCND1*, *UBN2*, *TTPAL*, *CACNG8*, *ORAI2*, and *XIAP*) that may be vital in AD and PSCI development.

In AD, Aβ deposition and tau neurofibrillary entanglement are classic pathological changes that lead to amyloid precursor deposition, autophagy, apoptosis, and mitochondrial dysfunction. Interestingly, post-stroke degenerative gene expression in hippocampal neurons cause similar pathological changes^58^, suggesting that gene expression patterns may be the source of mechanistic overlap between AD and PSCI. In particular, p53 regulation of miRNAs is responsive to stressors, thus influencing gene stability and protein function; both Aβ deposition and tau hyperphosphorylation in the CNS can be attributed to p53^59–62^. Indeed, the p53-miRNA interaction has been widely researched as a target for treating neurodegenerative diseases. Our study identified hsa-miR-548as-5p, hsa-miR-5186, hsa-miR-548ak, hsa-miR-147a, hsa-miR-6868-3p, and hsa-miR-449b-5p as part of the p53-related signaling pathway.

Research on other diseases and related signaling mechanisms have helped us understand the pivotal roles of several DE-miRNAs in the occurrence of AD and PSCI. First, hsa-miR-187-5p, hsa-miR-3157-5p, and hsa-miR-519c-3p—upregulated in the AD group—are implicated in the PI3K signaling pathway. This is a lipid kinase that participates in the intracellular signaling cascade, as well as cell silencing, survival, and growth under physiological and disease states^63,64^. Mesenchymal stem cell-derived exosomal miR-223 can protect neurons from apoptosis through protein tyrosine phosphatase (PTEN), which targets the PI3K/Akt pathway^65^. Additionally, hsa-miR-4655-5p and hsa-miR-3157-5p are involved in regulating mitogen-activated protein kinase (MAPKs) pathways. In mammals, MAPKs include c-Jun NH2-terminal kinase (JNK), p38 MAPK, and extracellular signal-regulated kinase (ERK). The pathway is implicated in cancer and neurodegenerative diseases through its regulation of cell proliferation, differentiation, and intrinsic immunity^66^. In particular, p38 MAPK affects microglia- and astrocyte-mediated neuroinflammation. This process has been linked to RNase III enzyme Drosha, which accelerates miRNA maturation. Evaluation of postmortem tissues from AD patients and AD rat models suggests that Aβ oligomers induce p38 MAPK-dependent phosphorylation of Drosha, leading to its bidirectional distribution between the neuronal cytoplasm and nucleus. Drosha overexpression can protect neurons from Aβ oligomeric-induced apoptosis^67^. Furthermore, MAPK can affect glucose metabolism in the CNS, promote catecholamine uptake, regulate neuronal growth, and alter neurotransmitter GABA expression via modulating insulin signal transduction pathways^68^. Triggering these processes can potentially improve cognitive function in patients with AD and PSCI.

Key genes were screened to further explore the mechanisms underlying AD and PSCI. The tumor suppressor *PTEN* is regulated by hsa-miR-519c-3p and mediates disease progression through activating the PI3K/serine-threonine kinase (AKT) signaling pathway. Pathological tau protein and intracellular cholesterol enrichment trigger premature *PTEN* activation, accelerating the microglia toxicity that occurs when synapses or neurons are exposed to phosphoserine apoptotic signals^69,70^. In vitro and in vivo AD models have confirmed that *PTEN* inhibition can effectively alleviate synaptic and cognitive damage^71^. In particular, the isoform PTENα can affect olfactory behavior in AD mice and regulate the endocytosis of olfactory bulb neurons through direct dephosphorylation of endocytosis proteins, a phenomenon also observed in patients with Parkinson's disease and DLB^72^. In addition to PTENα, PTEN-induced kinase 1 (PINK1) controls the specific clearance of dysfunctional or excess mitochondria through selective autophagy regulation^55^. PINK1 overexpression can reverse abnormal changes in mitochondrial dynamics, autophagy defects, and abnormal energy metabolism in the hippocampus. Other functions of PINK1 include upregulating antioxidant proteins to lower oxidative stress and inhibiting stress-induced neuronal apoptosis via the Nrf2 pathway^73^. In an AD mouse model, plasma PTEN-PDZ complex is relatively stable and crosses the BBB to become PTEN, resulting in synaptic protection and improved cognitive function. These two characteristics (plasma stability and BBB permeability) make the PTEN-PDZ complex a promising candidate for parenteral (intravenous and intramuscular) and naso-cerebral administration^74^.

Transcription factors regulate miRNA production in response to external stimuli and are especially active in the brain^75^. Under ischemia and hypoxia, transcription factors inactivate neurons within a few minutes, causing serious brain dysfunction. Sensitivity to damage increases with increasing age and greater impairment to the energy transport capacity of cells. In this study, *HIF1A* (regulated by hsa-miR-519c-3p) encodes the alpha subunit of hypoxia-inducible factor-1 (HIF-1). As a key transcription factor, HIF-1 is an important mediator of cellular and systemic homeostasis. HIF-1α upregulates BACE1 and γ-secretase in ischemic anoxic mouse models and primary neuron culture, inducing Aβ production during brain tissue hypoperfusion or hypoxia^76^. Likewise, the mTOR-HIF1α complex induces acute inflammation in microglia exposed to Aβ. Metabolic reprogramming from oxidative phosphorylation to glycolysis eventually causes abnormal metabolism, cytokine secretion, and phagocytosis^77,78^. In addition to its effect on Aβ, HIF-1α also influences tau neurofibrillary tangles under chronic hypoxia. In SD mouse hippocampal neurons after hypoxia treatment, an increase in HIF-1α is accompanied by tau protein hyperphosphorylation, along with a decrease in protein phosphatase 2A and leucin carboxymethyltransferase 1. These molecular changes are linked to cognitive impairment among SD mice and were verified in primary hippocampal neurons and C6/tau cells^79^. Thus, inhibiting HIF-1α may alleviate hypoxia-related cognitive impairment. Moreover, HIF1α could also cause remission of some PSCI symptoms, specifically irreversible white matter and neuronal damage. The mechanism involves HIF1α activation of the PKA pathway to regulate angiogenesis induced by vascular endothelial growth factor and erythropoietin^72,80^.

Another important target gene we identified here is the neuronal gene BMI1^81^. Whole-genome sequencing on samples from patients with late-onset AD revealed that BMI expression decreased, in conjunction with changes to Aβ and P-tau^82^. BMI1 inhibits the transcription of tau-associated tubulin, and knocking out BMI1 in neurons leads to Aβ secretion/deposition, P-Tau aggregation, and neurodegenerative changes^83^. A clinical cohort study comprising 1565 patients with AD found a significant correlation between BMI1 expression and Aβ1-42 levels in cerebrospinal fluid^83^. Taken together, available data strongly implicate BMI1 as a potential therapeutic target for AD.

The widespread use of miRNAs as a non-invasive screening method has revealed that a single miRNA or miRNA complex in plasma possesses differential diagnostic efficacy. A key obstacle in identifying relevant target miRNAs is the considerable heterogeneity in miRNA expression. Our study identified several miRNAs that are entirely novel or have only been minimally documented in a few publications associated with non-neurological disorders. Thus, little data are available on whether they can truly serve as biomarkers of PSCI or AD. In AD, bidirectional interactions between neuroinflammatory OS and the proteins Aβ and tau could also influence miRNA expression. Moreover, key miRNA synthesis enzymes such as Drosha, Dicer, and AGO2 are downregulated by stressful conditions such as hypoxia, causing aberrant miRNA expression that may interfere with diagnostic accuracy^84–86^.

One notable limitation of plasma miRNA sequencing is that it only reflects miRNA expression of the disease stage at sampling. While such data can be applied to differential diagnosis and prognosis, it obviously does not reflect miRNA expression patterns throughout the disease course. In this study, miRNA expression represented the post-symptomatic state because only clinically diagnosed participants were included. However, DE-miRNAs have considerable potential for diagnosis before requiring patients to undergo expensive and invasive examinations such as PET scans and lumbar punctures. Beyond an earlier diagnosis, DE-miRNA data also provides more information to clinicians regarding specific disease types, suggesting major benefits in addressing current shortcomings. To overcome the obstacles of heterogeneous miRNA expression, samples at different stages of disease progression are needed to identify common regulatory miRNAs that can then be therapeutically manipulated^87^.

Therapies based on miRNAs are currently in the form of mimics or inhibitors to influence downstream signaling pathways. However, further research is needed to determine optimal drug delivery in humans. In the future, anti-microRNA (AM) strategies could be implemented for the amelioration and clinical management of AD. This novel therapeutic approach restores multiple miRNA-regulated gene targets via the use of selectively stabilized AM species. Moreover, only a relatively small amount of miRNA families need to be modulated or neutralized to re-establish neuronal homeostasis in the AD-affected brain^88^.

In conclusion, through high-throughput sequencing and bioinformatics, we identified novel miRNAs, their key target genes, and highlighted potential mechanisms of their involvement in AD and PSCI. Importantly, our findings provided an empirical basis for using miRNAs as peripheral biomarkers and therapeutic targets.

## Methods

### Participants

The study selected 18 patients admitted to the Department of Neurology at the First Hospital of Jilin University between 2021 and 2022. Based on etiology and MoCA, patients were divided into three groups (n = 6 per group): AD, PSCI, and post-stroke non-cognitive impairment (PSNCI). Six normal controls (NC) were also included.

Patients were included in the PSCI or PSNCI group if they were 50–80 years of age, met the WHO diagnostic criteria for acute ischemic stroke (AIS), and could complete the relevant scale assessment. They were further subdivided into PSNCI if MoCA ≥ 22 and PSCI if MoCA < 22^89^. Patients were included in AD group if they met the NINCDS-ADRDA diagnostic criteria and confounding factors were excluded. Finally, spouses of the patients were included as NC, matched by age, sex, education and previous medical history.

Individuals were excluded if they exhibited other CNS conditions that cause cognitive impairment, including metabolic encephalopathy, Parkinson's syndrome, Huntington's disease, subdural hematoma, normal cranial pressure hydrocephalus, brain tumors, and brain trauma. Displaying other systemic conditions linked to cognitive decline also resulted in exclusion; these included hypothyroidism, vitamin (B12, folic acid, niacin) deficiency, severe anemia, liver or renal insufficiency, hyponatremia, hypocalcemia, neurosyphilis, HIV infection, and alcohol/drug abuse. Finally, disorders or any other characteristics that negatively impacted the ability to perform tasks and scale operations led to exclusion. These included mental disorders (e.g., depression and schizophrenia), nuclear magnetic contraindications, refusal of blood collection, serious diseases of vital organs, and sensory impairments (e.g., blindness, aphasia, deafness).

### Data collection

Patient age, sex, education, other demographics, and related medical history (e.g., hypertension, diabetes, coronary heart disease) were collected. Professional neurologists conducted all neuropsychological assessments of participants to obtain relevant baseline data. After an 8-h fasting period, peripheral venous blood samples were collected, and plasma was separated for storage at − 80 °C.

### Statistical analysis

Statistical analyses were performed in IBM SPSS Statistics version 25 (IBM, Armonk, NY, USA) and R version 4.3.2 (R Foundation for Statistical Computing, Vienna, Austria). Sociodemographic and neuropsychological characteristics were subjected to descriptive analyses. Continuous variables were presented as mean ± SD or median (interquartile range), and categorical variables were presented as frequencies (percentages). Between-group differences were compared with chi-square tests, one-way ANOVA, or Kruskal–Wallis rank sum test. Significance was set at *P* < 0.05.

### Microarray assay of miRNA

Human tissue specimens were collected from the Department of Biobank, Division of Clinical Research, for RNA extraction using TRIzol reagent (Invitrogen, Carlsbad, CA, USA). MiRNA sequencing was performed using an Illumina NextSeq 500 sequencing platform (San Diego, CA, USA). Double-stranded cDNA was synthesized using the dUTP method and high-fidelity PCR polymerase to ensure strand specificity. Constructed libraries were assessed in an Agilent 2100 Bioanalyzer and subjected to quantitative real-time PCR (qRT-PCR).

Based on quantification results, libraries were mixed in equal amounts and used for sequencing. Single-stranded DNA (1.8 pM) generated from denaturing with 0.1 M NaOH were loaded onto the reagent cartridge. Sequencing was performed for 50 cycles on a NextSeq system using the NextSeq 500/550 V2 kit (#FC-404-2005, Illumina). The Solexa pipeline v1.8 (Off-Line Base Caller v1.8) was used for image analysis and base calling. Sequencing quality was examined with FastQC.

Expression profiles of miRNAs were calculated based on mapped read counts. Differentially expressed miRNAs (*P* < 0.05, |log2FC|≥ 1) were screened in R package *edgeR*^90^, then matched to reference sequences in miRDeep2^91^. The expression of miRNAs was represented as counts per million mapped reads (CPM) after transformation.

### Bioinformatics analysis

Sequencing cluster maps of the three groups were generated with R (v4.3.2) packages *ggplot2* and k-means clustering. To select DE-miRNA, volcano plots were created with *ggplot2*. Next, DE-miRNA target genes were predicted with reference to three databases: MiRDIP (http://ophid.utoronto.ca/mirDIP/index.jsp)^92^, miRNet^93^ (https://www.mirnet.ca/), and TargetScan Human (https://www.targetscan.org/vert_80/)^94^. Hits in all three databases comprised the final list of target genes.

Functional analyses with GO^95^ and KEGG^96^ were visualized using R packages *ClusterProfiler* and *ggplot2*. Significant enrichment (P < 0.05 with Benjamin-Hochberg corrections) was determined with Fisher’s exact test. Next, the PPI network of target genes was generated in STRING (https://cn.String-db.org/) and visualized in Cytoscape (v3.9.1)^97^. Target genes were processed in MCODE (node score cutoff: 0.2, K-Core: 2, Max. Depth: 100) and CytoHubba (MCC algorithm) to identify key genes (those that passed both sets of conditions). Finally, a network of signaling pathways involving DE-miRNAs and their key genes was constructed.

### Ethical approval and informed consent

All methods were performed in accordance with the relevant guidelines and regulations (For ex. Declaration of Helsinki). The studies involving human participants were reviewed and approved by the ethics committee of The First Hospital of Jilin University (ethics number: 19K023-003). All subjects provided written informed consent to participate in this study. The sequencing information of all participants has been filed with the China Center for Human Genetic Resources Management.

### Supplementary Information

Supplementary Legends.

Supplementary Table S1.

Supplementary Table S2.

Supplementary Table S3.

Supplementary Table S4.

Supplementary Table S5.

Supplementary Table S6.

Supplementary Table S7.

Supplementary Table S8.

Supplementary Table S9.

Supplementary Table S10.

## Data Availability

All microarray data used in this study are publicly available through the Gene Expression Omnibus (GEO) database, accession numbers: GSE218605 (https://www.ncbi.nlm.nih.gov/geo/). The raw sequence data reported in this paper have been deposited in the Genome Sequence Archive (Genomics, Proteomics & Bioinformatics 2021) in the National Genomics Data Center (Nucleic Acids Res 2022), China National Center for Bioinformation/Beijing Institute of Genomics, Chinese Academy of Sciences (GSA-Human: HRA003714), which are publicly accessible at https://ngdc.cncb.ac.cn/gsa-human^98,99^.
