# Supplementary material for: Biomarker profiling to determine clinical impact of microRNAs in cognitive disorders
Source: Sci Rep. 2024 Apr 9;14:8270. doi: 10.1038/s41598-024-58882-2 (PMC11004146; doi:10.1038/s41598-024-58882-2)
Supplement: Supplementary file 1 — Supplementary Legends. [file 41598_2024_58882_MOESM1_ESM.docx]

**Supplementary tables：**

1. Table S1. Medical history in each group
2. Table S2. All Differentially Expressed miRNA
3. Table S3. Enrichment GO of AD vs NC
4. Table S4. Enrichment KEGG of AD vs NC
5. Table S5. Enrichment GO of AD vs PSCI
6. Table S6. Enrichment KEGG of AD vs PSCI
7. Table S7. Enrichment GO of PSCI vs PSNCI
8. Table S8. Enrichment KEGG of PSCI vs PSNCI
9. Table S9. Venn of miRNAs of three groups
10. Table S10. Venn of target genes of three groups
