# Supplementary material for: Biomarker profiling to determine clinical impact of microRNAs in cognitive disorders
Source: Sci Rep. 2024 Apr 9;14:8270. doi: 10.1038/s41598-024-58882-2 (PMC11004146; doi:10.1038/s41598-024-58882-2)
Supplement: Supplementary file 2 — Supplementary Table S1. [file 41598_2024_58882_MOESM2_ESM.docx]

**Table S1.** Medical history in each group

|  | AD | PSCI | PSNCI | NC | P |
| --- | --- | --- | --- | --- | --- |
| age | 55.5（53.7-68.8） | 62.0（57.0-72.0） | 57.5（53.8-65.3） | 52.5（50.0-57.3） | 0.125 |
| female | 3（50%） | 2（33.3%） | 0（0.0%） | 5 (83.3%) | **0.035** |
| education | 10.5（9.0-15.0） | 9.0（9.0-9.8） | 10.5（9.0-12.0） | 12.0（9.0-16.0） | 0.396 |
| hypertension | 3（50.0%） | 3（50.0%） | 3（50.0%） | 1（16.7%） | 0.725 |
| diabetes | 1（16.7%） | 2（33.3%） | 2（33.3%） | 0（0.0%） | 0.695 |
| heart disease | 2（33.3%） | 0（0.0%） | 1（16.7%） | 1（16.7%） | 0.878 |
| history of stroke | 2（33.3%） | 0（0.0%） | 0（0.0%） | 0（0.0%） | 0.217 |
| history of smoking | 4（66.7%） | 3（50.0%） | 3（50.0%） | 0（0.0%） | 0.119 |
| history of drinking | 3（50.0%） | 1（16.7%） | 4（66.7%） | 1（16.7%） | 0.277 |

Table S1 presents a comparative analysis of basic information like age, sex and education and medical history in each group.
